# The differences between insulin glargine U300 and insulin degludec U100 in impact on the glycaemic variability, arterial stiffness and the lipid profiles in insulin naïve patients suffering from type two diabetes mellitus – outcomes from cross‐over open-label randomized trial

**DOI:** 10.1186/s12902-021-00746-1

**Published:** 2021-04-29

**Authors:** Pavle Vrebalov Cindro, Mladen Krnić, Darko Modun, Božo Smajić, Jonatan Vuković

**Affiliations:** 1 Department of Gastroenterology, University Hospital Split, Spinčićeva 1, 21000 Split, Croatia; 2 Department of Endocrinology, University Hospital Split, Šoltanska 1, 21000 Split, Croatia; 3 Department of Pharmacy, University of Split School of Medicine, Šoltanska 2, 21000 Split, Croatia; 4 Department of Pathophysiology, University of Split School of Medicine, Šoltanska 2, 21000 Split, Croatia; 5 Medical Student, University of Split School of Medicine, Šoltanska 2, 21000 Split, Croatia

**Keywords:** Diabetes mellitus type 2, Degludec, Glargine U300, Glucose variability, Arterial stiffness, Lipids

## Abstract

**Background and aims:**

Diabetes mellitus type two is one of the major cardiovascular risk factors. Treatment of diabetes can reduce this risk, but the treatment options differ a lot in their risk-reducing capabilities. We compared the impact of insulin degludec (IDeg-100) and insulin glargine U300 (IGlar-300) on cardiovascular risk parameters - glycaemic variability (GV), arterial stiffness and lipid parameters - in insulin naive patients with DMT2.

**Methods:**

To 23 individuals who previously had uncontrolled DMT2 on two or more oral antidiabetic drugs, IGlar-300 and IDeg-100 were applied for 12 weeks and then switched in a cross over design manner. Prior and after of each insulin phase, we analysed biochemical parameters,7-point SMBG profile over three days and arterial stiffness which was assessed indirectly by measuring the augmentation index (AIx) on the principles of applanation tonometry.

**Results:**

There were no significant differences between IGlar-300 and IDeg-100 regarding reduction of mean glucose values and coefficient of variation (CV). Both insulins insignificantly reduced AIx for standardised pulse of 75 beats/min and without differences between them. IGlar-300 and IDeg-100 reduced triglycerides and increased HDL with no significant difference between the two insulins. IGlar-300 increased the total cholesterol level and IDeg-100 decreased total cholesterol, but without statistically significant difference. IGlar-300 increased LDL level by 0.508 mmol/L and IDeg-100 decreased LDL by 0.217 mmol/L, with statistically significant difference (*p* = 0.0215).

**Conclusions:**

This study did not show significant difference between IGlar-300 and IDeg-100 regarding glycaemic parameters and augmentation index using the same dose of 0.2 IU/kg for both insulins, but it has revealed possible differences in impact on lipid profile.

**Trial Registration:**

Clinicaltrials.gov, NCT04692415. Retrospectively registered on December 31th 2020.

**Supplementary Information:**

The online version contains supplementary material available at 10.1186/s12902-021-00746-1.

## Background

It is well known that the level of glucose control in patients with diabetes mellitus type two (DMT2) is associated with cardiovascular outcomes [1]. Much less is known about the impact of glycaemic variability (glycaemic fluctuations from peaks to nadirs) on vascular function in contrast to hyperglycaemia *per se*. Although the pathogenesis of atherosclerosis is mediated through multiple and complex mechanisms, including lipoprotein abnormalities [2], certain studies have shown that oscillating glucose can have more deleterious effects than constant high glucose on endothelial function (directly) and oxidative stress [3]. Chronic sustained hyperglycaemia and glycaemic variability both contribute to diabetic cardiovascular complications causing excessive protein glycation and oxidative stress [4] (which, then, worsen endothelial function indirectly), but glycaemic variability is more specific in producing effect on oxidative stress [5], as both postprandial glucose increments and interprandial glucose decrements activate the oxidative stress [6]. Arterial stiffness represents a prognostic marker of cardiovascular disease in diabetic patients and also an indirect measure of target organ damage [7]. It can be measured indirectly through augmentation index (AIx), which is an indirect marker for arterial stiffness and a direct measure of wave reflection [8].

Insulin therapy improves insulin action on glucose metabolism and decreases AIx [9] as insulin possesses both vasodilatory and sympathomimetic activities [10]. Insulin therapy also activates lipid metabolism and shows anti-atherogenic effects, contributing additionally to the decrease of arterial stiffness [11].

Nowadays, long-acting insulin analogues in combination with oral antidiabetic therapy represent the most common initial insulin therapy regimen. They exhibit longer duration, flatter action profiles, lower risk of severe and nocturnal hypoglycaemia and less glycaemic variability, compared to older basal insulins [12, 13]. Along with the emerging of the new generation of insulin analogues (degludec and glargine U300) came the comparisons between these two insulins, mostly in terms of incidence of hypoglycaemia [13–16], and pharmacokinetic/pharmacodynamic (PK/PD) characteristics [13, 17, 18].

To date, there is little data and head-to-head comparisons of IDeg-100 and IGlar-300 considering the impact on the glycaemic variability, arterial stiffness and lipid profile in type two diabetes mellitus (DMT2). In this study, we aimed to compare the impact of insulin degludec and insulin glargine U300 in insulin naive patients suffering from diabetes mellitus type two on these parameters.

## Materials and methods

### Study protocol and population

**Fig. 1:**
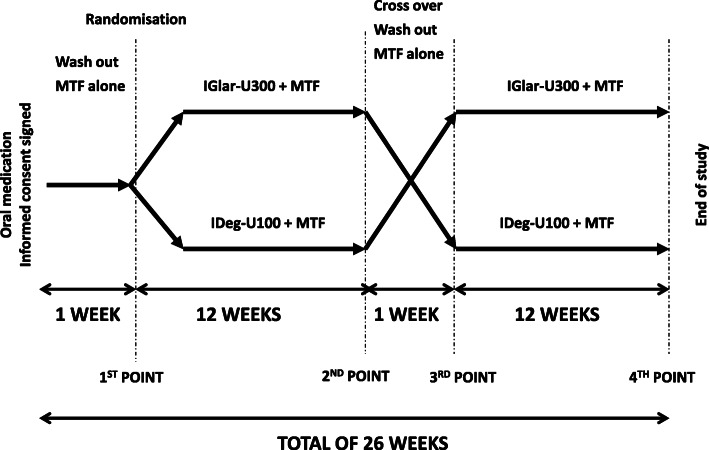
The study protocol

This randomized, open-label, crossover study was conducted in accordance with the Declaration of Helsinki and approved by the Ethics Committee of the University of Split School of Medicine (number 2181-198-03-04-17-0045). All subjects gave written consent prior to their participation in the study. Between December 2018 and May 2019, we recruited the total of 25 outpatient and inpatient insulin naïve patients with T2DM who were uncontrolled on two or more oral antidiabetic drugs and assigned them to either degludec insulin or glargine U300 insulin combined with metformin. All patients were recruited and treated at University Hospital Split, Croatia. All patients finished the study, but only 23 were analysed, as two of them did not perform SMBG as requested. Basal characteristics of the participants studied are shown in Table 1. The protocol of the study is shown in Fig. 1. The study adheres to CONSORT guidelines (Additional file 1 – CONSORT flow diagram).

**Table 1:** Clinical characteristics of patients

Total number	23 (16 men)	
Parameter	Mean	(± SD)
Age (years old)	57.45	6.89
Duration of diabetes (years)	9.71	4.95
Body weight (kg)	89.37	14.30
Body height (cm)	175.35	9.64
Body mass index (kg/m2)	29.30	3.83
Waist circumference (cm)	103.48	8.63
HbA1c (%)	9.67	1.67
Fasting glucose (mmol/L)	12.98	4.51
Serum creatinine (µmol/L)	67.22	13.18
Serum uric acid (µmol/L)	307.57	62.10
Total cholesterol (mmol/L)	5.07	1.10
HDL	1.08	0.23
LDL	3.02	1.00
TG	2.29	0.83

*SD* standard deviation; *HDL* high density lipoprotein; *LDL* low density lipoproteinl; *TG* triglycerides

Subjects who were eligible for the study had fulfilled all of the following inclusion criteria: history of DMT2 for at least 1 year, aged between 45 and 65 years (women obligatory in postmenopausis), uncontrolled glycaemia on two or more oral antidiabetic drugs, no prior use of insulin, HbA1c ≥ 7.5 %, receiving statins (if not on statins, they were put on it), not on antiaggregant therapy (if on antiaggregants, they were temporarily excluded from therapy).

Exclusion criteria was: the presence of malignant disease, chronic liver disease, renal impairment with creatinine clearance < 60 ml/s, severe cardiovascular disease or history of cardiovascular incidents (stroke, myocardial infarction, peripheral amputation), rheumatic and autoimmune diseases and the usage of glitazones or anticoagulant therapy.

All subjects were asked to avoid the consumption of coffee, coca-cola and similar beverages, wine and vitamin supplements, especially within several days before each control point. Patients were also asked to avoid intensive physical activity up to two days before each control point.

At baseline, all participants were discontinued with their previous therapy and entered wash-up period which lasted for seven days and in which they were given metformin alone (2 g/day). After wash-up period, they were randomized alternately by investigators (1:1 ratio) to first receive either IDeg-100 in dose of 0.2 IU/kg or IGlar-300 in the same dose of 0.2 IU/kg according to the order they were included in the study and started with phase one in which they received either IDeg-100 and metformin or IGlar-300 and metformin for 12 weeks. Phase one was followed by second wash-up period in which patients received metformin alone again for seven days. In phase two, which also lasted for 12 weeks, participants were switched from IDeg-100 to IGlar-300 and conversely, with metformin continued. The initial dose of both insulins was 0.2 IU/kg. We did not up-titrate the dose of insulin during the study period to avoid hypoglycaemia in the first place as hypoglycaemia could significantly influence the results [19–21].

At the beginning and the end of each phase blood samples for the analysis of standard biochemical parameters were collected (control points 1–4) and augmentation index was measured. In three consecutive days prior to each control point, patients performed 7-point SMBG (Self-Monitoring of Blood Glucose) profile [22].

### Glucose measurement

To standardize results, all patients received standard Bionime GM550 glucose meter. They were asked to regularly control blood glucose one or two times per day during the entire study, and, in three consecutive days prior to each control point, to perform the 7-point SMBG profile. The 7-point blood glucose profile consisted of seven measurements: (1) - before breakfast, (2) − 2 h after breakfast, (3) - before lunch, (4) − 2 h after lunch, (5) - before dinner, 6.- 2 h after dinner and 7. - before sleeping. Glucose variability was determined by calculating mean glycaemia, standard deviation and coefficient of variation in each control point [22, 23].

### Standard laboratory measurement

Total cholesterol, HDL, LDL, triglycerides and other basic biochemical laboratory values were determined by automatic analyzer Olympus AU 600 (Olympus Michima Co. LTD, Shizouka, Japan) and enzymatic laboratory kit.

### Measurements of arterial stiffness

The augmentation index (AIx) corresponds to the pressure difference between initial systolic (P1) and reflected wave (P2) in a relation to the pulse pressure (PP). It is calculated on the basis of the formula: AIx (%) = [(P2-P1)/PP] × 100 [24]. The AIx represents an indirect marker for arterial stiffness and a direct measure of wave reflection [25, 26]. In this study we used SphygmoCor (AtCor Medical, Sydney, Australia) which allow non-invasive measurement of AIx on radial artery using strain gauge transducer placed on the tip of a pencil-type tonometer. This method is based on the principle of applanation tonometry [27].

### Statistical analysis

The number of subjects to include in the protocol was selected according to the previous available literature [5, 28]. Statistical analyses were performed using Statistica 6.0 (StatSoft Inc., Tulsa, USA). Two-way ANOVA for repeated measures was used to evaluate IDeg-100 and IGlar-300 dependent changes in plasma glucose levels, coefficient of glucose variation, lipid levels and AIx.

## Results

All patients (25 in total, 7 females) completed the trial, 23 were analysed. No unexpected events, harms or unintended effects of therapy were observed. Their mean basal values are given in Table 1.

### Glycaemic variability

**Fig. 2:**
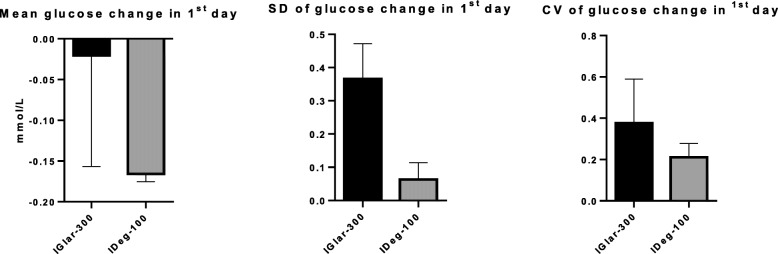
Mean glucose change, SD and CV in the1st day. SD = standard deviation, CV = coefficient of variation, IGlar-300 = insulin glargine U300, IDeg-100 = insulin degludec U100.

On the first of three consecutive days of 7-point SMBG, performed at the end of the observed period, IGlar-300 and IDeg-100 reduced mean glucose values by 0.02 and 0.16 mmol/L, respectively, which was statistically insignificant (*p* = 0.06), and without significant difference between the two insulins (*p* = 0.17) (Fig. 2.).

Standard deviation (SD) of glucose excursions was 0.36 for IGlar-300 and 0.06 for IDeg-100 which was insignificant (*p* = 0.2) and in comparison of the two insulins there was no statistically significant difference (*p* = 0.07) (Fig. 2.)

The coefficient of glucose variation (CV) on the first day was 0.37 (37 %) for IGlar-300 and 0.21 for IDeg-100 which was statistically insignificant (*p* = 0.22). When compared, CV for these two insulins was not significantly different (*p* = 0.20) (Fig. 2.).

**Fig. 3:**
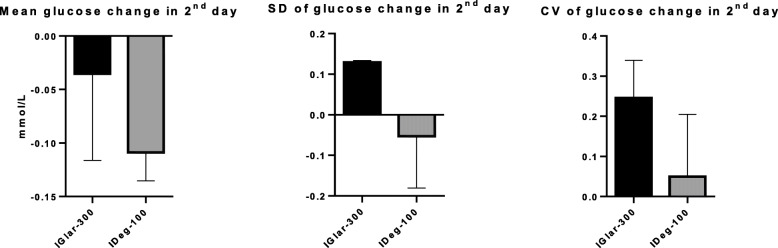
Mean glucose change, SD and CV in the 2nd day. SD = standard deviation, CV = coefficient of variation, IGlar-300 = insulin glargine U300, IDeg-100 = insulin degludec U100.

On the second of the three days of 7-point SMBG, performed at the end of the observed period, IGlar-300 and IDeg-100 reduced mean glucose by 0.03 and 0.10 mmol/L, respectively, which was statistically insignificant (*p* = 0.08), and without significant difference between the two insulins (*p* = 0.07) (Fig. 3.).

Second day SD of glucose excursions was 0.12 for IGlar-300 and − 0.05 for IDeg-100 which was insignificant (*p* = 0.19) and when we compared the SD of the two insulins there was no significant difference (*p* = 0.17) (Fig. 3.).

The CV for the second day was 0.24 (24 %) for IGlar-300 and 0.04 for IDeg-100 and that was statistically insignificant (*p* = 0.20). When compared, CV for the two insulins was no significantly different (*p* = 0.08) (Fig. 3.).

**Fig. 4:**
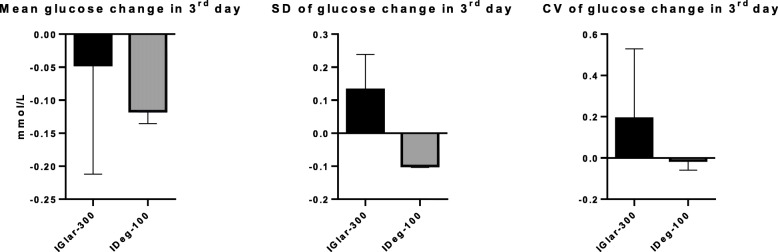
Mean glucose change, SD and CV in the 3rd day. SD = standard deviation, CV = coefficient of variation, IGlar-300 = insulin glargine U300, IDeg-100 = insulin degludec U100.

On the third (last) day of the SMBG, the insulins reduced mean glucose levels by 0.04 (IGlar-300) and by 0.11 mmol/L (IDeg-100) which was statistically insignificant (*p* = 0.08), again without significant difference between the two insulins (*p* = 0.20) (Fig. 4.).

SD of glucose concentrations on third day was 0.13 for IGlar-300 and − 0.09 for IDeg-100 which was insignificant (*p* = 0.18) and comparison of SD of the two insulins revealed no statistical difference (*p* = 0.14) (Fig. 4.).

The 3rd day CV was 0.19 for IGlar-300 and − 0.01 for IDeg-100 which was statistically insignificant with *p* = 0.16. When compared, CV for these two insulins was no statistically different (*p* = 0.54) (Fig. 4.).

### Augmentation index

**Fig. 5:**
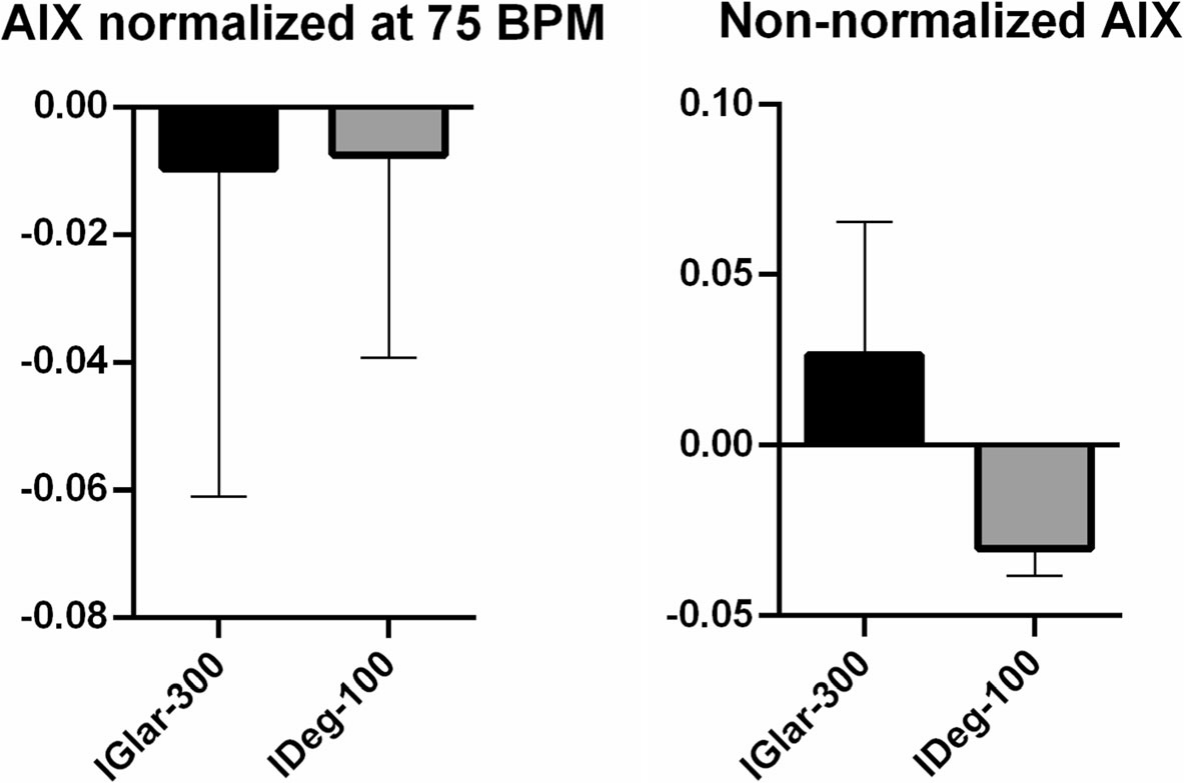
Mean change in augmentation index. AIX = augmentation index, BPM = beats per minute, IGlar-300 = insulin glargine U300, IDeg-100 = insulin degludec U100.

Both insulins reduced AIx for standardised pulse of 75 beats per minute. IGlar-300 and IDeg-100 decreased AIx by 0.009 and 0.007 %, respectively. This was statistically insignificant (*p* = 0.059 with 95 % CI -0.127 to 0.122). There was no significant difference between the two insulins (*p* = 0.11, 95 % CI -0.132 to 0.367) (Fig. 5.).

AIx measured for “non-normalized” pulse was increased by 0.026 % for IGlar-300, while IDeg-100 reduced it by 0.03 %, which was insignificant (*p* = 0.06). When compared, there was no difference in AIx between the two insulins (*p* = 0.06) (Fig. 5.).

### Lipids

**Fig. 6:**
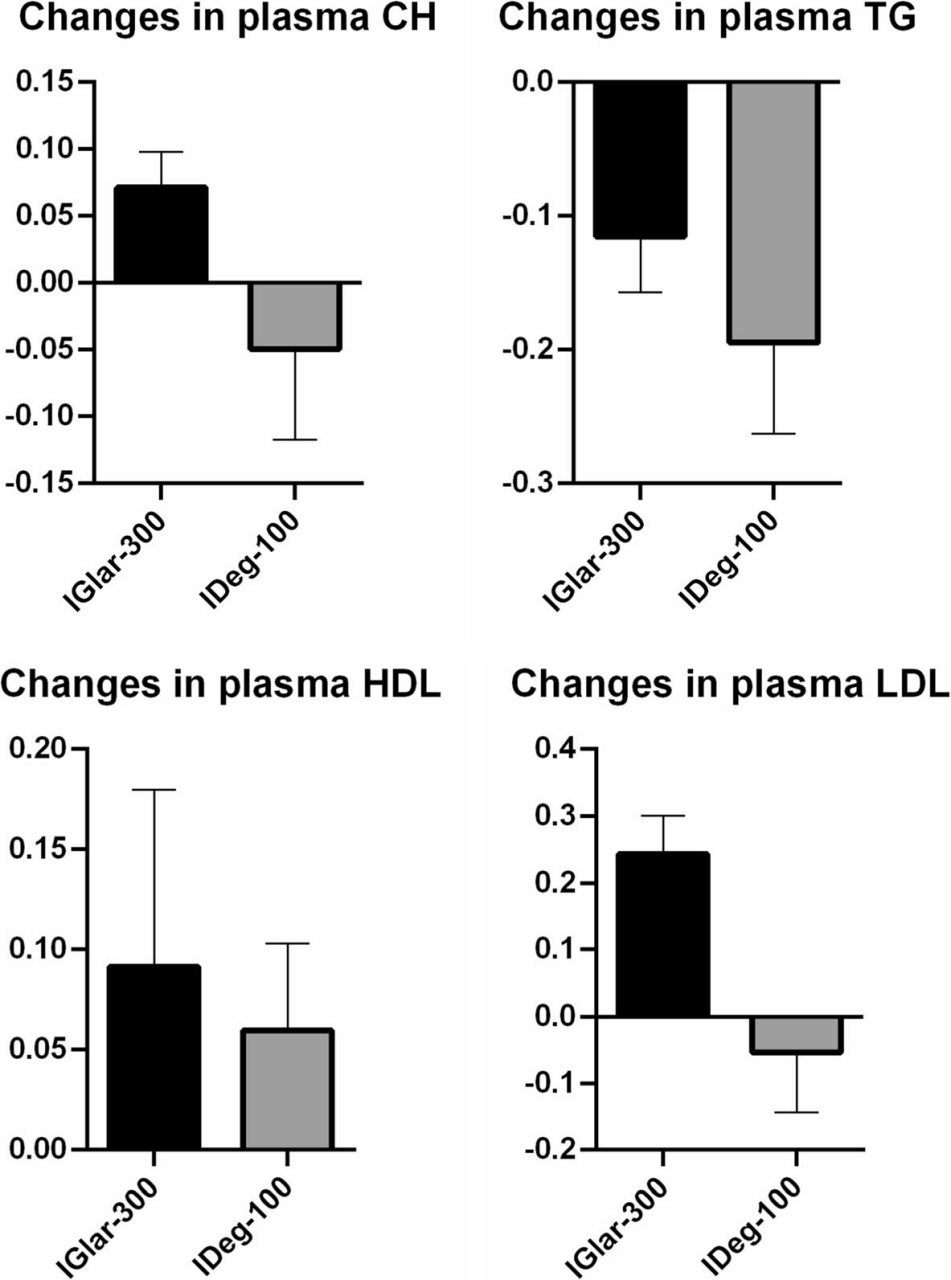
Changes in plasma lipids. The only statistically significant difference between two insulins was in the impact on LDL. TG = triglycerides, CH = total cholesterol, HDL = high density protein, LDL = low density protein, IGlar-300 = insulin glargine U300, IDeg-100 = insulin degludec U100. The results are depicted as derivations of absolute values and represented as percentage of change.

IGlar-300 and IDeg-100 reduced triglycerides by 0.417 mmol/L and 0.595 mmol/L respectively, with no significant difference (*p* = 0.4275). IGlar-300 increased the total cholesterol level by 0.317 mmol/L and IDeg-100 decreased total cholesterol by 0.295 mmol/L, but without statistically significant difference (*p* = 0.0813). Both insulins increased HDL level – IGlar-300 by 0.1 mmol/L, and IDeg-100 by 0.065. There was no significant diffference in impact on HDL (*P* = 0.4015). IGlar-300 increased LDL level by 0.508 mmol/L and IDeg-100 decreased LDL by 0.217 mmol/L, with statistically significant difference (*p* = 0.0215) (Fig. 6).

## Discussion

There is much convincing evidence that uncontrolled DMT2 is associated with an increased risk of arterial stiffness and, thus, of cardiovascular complications in further life [29]. However, it is not clear in which proportion different glycaemic parameters contribute to the development of cardiovacular complications. Some studies suggest that both fasting and postprandial hyperglycaemia are the main factor leading to an increase in stiffness of especially intermediate-sized arteries in DMT2 [30, 31]. In another study (albeit on patients with type 1 diabetes mellitus), authors found the same impact of postprandial hyperglycaemia, but they emphasized that the increase in arterial stiffness was not associated with glycaemic variability [32]. On the other side, there are data suggesting that oscillating glucose can have more deleterious effects than constant high glucose on endothelial function and oxidative stress [3, 28]. Also in patients with DMT2, fasting plasma glucose (FPG), postprandial plasma glucose (PPG) and HbA1c showed a positive association with CAVI (cardio-ankle vascular index) and baPWV (brachial-ankle pulse wave velocity), both markers of arterial stiffness, although not equivalent [33].

The impact of glucose variability on oxidative stress is relatively well established [3, 5, 21, 34, 35], but as the oxidative stress itself leads to the development of atherosclerosis through multiple mechanisms, it is a logical conclusion that GV should result also in the increase of arterial stiffness. Some of those mechanisms are increased formation of advanced glycation end-products (AGEs), increased expression of the receptors for AGEs, polyol pathway and hexosamine pathway. Also, oxidative stress negatively influences the anti-atherosclerotic endothelial enzymes, leading in this way to defective angiogenesis in response to ischemia and activation of proinflammatory and epigenetic mechanisms after the normalization of glycaemia (“hyperglycaemic memory”) [36].

Moreover, one of the mechanisms contributing to the development of atherosclerosis is diabetic dyslipidaemia, characterised by increased triglycerides and LDL and decreased HDL [37]. Circulating molecules of LDL do not participate directly in atherosclerosis development, but by structural modification of its apoB act as a ligand for macrophages in the arterial wall triggering foam cell formation and initiating atherosclerosis. Glycated LDL is doubled even in patients with well-controlled diabetes and is susceptible to oxidation and have atherogenic potential. Insulin therapy and glycaemic control increase the action of lipoprotein lipase and thus the HDL production. However, HDL may be dysfunctional in diabetic patients and glycation is shown to reduce the sphingosine-1-phosphate content of HDL influencing in that way its ability to act protectively during the oxidative stress [38].

Earlier studies demonstrated that insulin modify lipids by activating the enzyme adipose tissue lipoprotein lipase. This induces the clearance of very low-density lipoprotein (VLDL) particles and chylomicrons from the serum with consequent transportation of fatty acids to the fat tissue. Insulin also affects adipocytes by boosting triglyceride synthesis and the skeletal muscles by lowering the activity of lipoprotein lipase. The latter can intercept the accumulation of lipids in the muscles. Finally, insulin hinders hormone sensitive lipase in fat tissue, decreasing lipolysis in that way. The total result of insulin impact on the metabolism of the lipids is a decrease of circulating triglycerides and triglyceride-rich lipoprotein concentration [39].

There are very few studies depicting the impact of specified insulin formulations on lipid profiles.

As the induction of insulin in the treatment of diabetic patients has shown also a beneficial effect on glucose variability and arterial stiffness [9, 10], the direct and simultaneous evaluation of the glycaemic variability, lipid status and arterial stiffness following the insulin induction was the leading idea of the present study.

Furthermore, as insulin degludec and glargine U300 came into the market practically at the same time, the comparison of these two last-generation basal insulin analogues became unavoidable. As it is stressed in the Introduction, there are a number of studies comparing degludec and glargine U300, but not in the manner designed in this study.

The results of our study showed significant difference between these two insulins in LDL concentrations with unexpected failure of IGlar300 to reduce it. Although without statistical significance, there were signals that there could be more differences in other lipid parameters. These results should be confirmed in the studies with much larger number of subjects, longer duration and higher doses of insulins. Comparison with other basal insulins, and even short acting insulins, should be performed. Possible mechanisms through which potential differences occur, also need to be explained. Perhaps the different PK/PD profiles of different insulins can play a role in the degree of LDL receptor activation [40] or in plasma levels of cholesteryl ester transfer protein (CETP) which is shown to be up-regulated by insulin analog initiation therapy causing anti-atherogenic effects by increasing HDL-large and decreasing LDL-3 and LDL-4 subfractions [11].

The present study did not show significant differences in impact of IDeg100 and Iglar300 on glycaemic variability and arterial stiffness using the same dose of 0.2 IU/kg for both insulins. If we neglect the possibility that these two insulins simply are not significantly different, still there are several possible reasons for the lack of the difference.

Firstly, we had a relatively small number of subjects. Secondly, three months of exposure to each insulin could be regarded as relatively short period [9, 41], although some studies succeeded to demonstrate an acute beneficial effect of a single insulin injection on pulse wave velocity (PWV) [10]. Thirdly, the usage of 7-point SMBG profile instead of CGMS (continuous glucose monitoring system) or Flash monitoring system represent the certain limitation as well. Finally, probably the most important limitation of the present study was the relatively small dose of the insulin which prevented the full expression of action of both insulins.

We applied both insulins in the standard initiating dose of 0.2 IU per kg of body weight. As possible hypoglycaemia could have a strong impact on the results of glycaemic variability and oxidative stress, we decided not to up-titrate the dose to the end of observed period. Several studies have shown that hypoglycaemia can decrease nitric oxide and cause the “reperfusion like” effect, influencing the level of oxidative stress in that way [19–21]. Although some studies showed no difference between IDeg-100 and IGlar-300 regarding the incidence of hypoglycaemia [15, 16], according to Tibaldi et al., IDeg-100 showed lower number of hypoglycaemic events versus IGlar-300 [14]. That also could influence the results, contributing to degludec side. Somewhat greater potency of IDeg-100 is also reported, thus titration to target would probably lead to the differences in final doses and, consequently, make the comparison more difficult [15, 18]. However, the small dose of insulin used in this study remains the most probable cause of the absence of more pronounced results.

## Conclusions

To conclude, in this setting, IDeg-U100 and IGlar-U300 showed no significant difference in impact on the glycaemic variability and arterial stiffness using the same dose of 0.2 IU/kg for both insulins. We noticed significant difference on LDL levels with signals for potential differences in other lipid parameters. Thus, further investigations are needed to either confirm or oppose these findings.

## Supplementary Information

**Additional file 1.**

## Data Availability

The datasets used and/or analysed during the current study are available from the corresponding author on reasonable request.

## Abbreviations

IDeg-100: Insulin degludec U100
IGlar-300: Insulin glargine U300
DMT2: Diabetes mellitus type two
GV: Glycaemic variability
SMBG: Self-monitoring of blood glucose
AIx: Augmentation index
CV: Coefficient of variation
HDL: High density lipoprotein
LDL: Low density lipoprotein
PK/PD: Pharmacokinetic/pharmacodynamic
HbA1c: Glycated haemoglobin
TG: Triglycerides
SD: Standard deviation
IU: International units
BPM: Beats per minute
CH: Total cholesterol
FPG: Fasting plasma glucose
CAVI: Cardio-ankle vascular index
baPWV: Brachial-ankle pulse wave velocity
AGEs: Advanced glycation end-products
VLDL: Very low-density lipoprotein
CETP: Cholesteryl ester transfer protein
PWV: Pulse wave velocity
CGMS: Continuous glucose monitoring system
