# Supplementary material for: The differences between insulin glargine U300 and insulin degludec U100 in impact on the glycaemic variability, arterial stiffness and the lipid profiles in insulin naïve patients suffering from type two diabetes mellitus – outcomes from cross‐over open-label randomized trial
Source: BMC Endocr Disord. 2021 Apr 29;21:86. doi: 10.1186/s12902-021-00746-1 (PMC8082786; doi:10.1186/s12902-021-00746-1)
Supplement: Supplementary file 1 — Additional file 1. [file 12902_2021_746_MOESM1_ESM.doc]

**
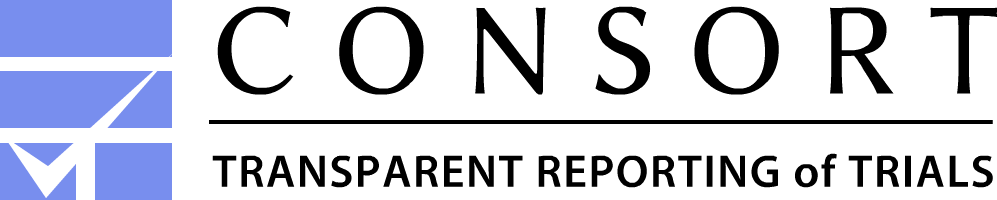
**

**CONSORT 2010 Flow Diagram - The differences between Insulin Glargine U300 and Insulin Degludec U100 in impact on the glycaemic variability, arterial stiffness...**

**Allocation (cross-over design)**

**Analysis**

**Follow-Up**

**Enrollment**

Assessed for eligibility (n=27)

Excluded (n=2)

♦  Not meeting inclusion criteria (n=1)

♦  Declined to participate (n=1)

♦  Other reasons (n=0)

Analysed (n=23)
♦ Excluded from analysis (give reasons) (n=2 ) – did not perform SMBG in all points as requested

Lost to follow-up (give reasons) (n=0)

Discontinued intervention (give reasons) (n=0)

Allocated to intervention (n=25)

♦ Received allocated intervention (n=25)

♦ Did not receive allocated intervention (give reasons) (n=0)

Lost to follow-up (give reasons) (n=0)

Discontinued intervention (give reasons) (n=0)

Allocated to intervention (n=25)

♦ Received allocated intervention (n=25)

♦ Did not receive allocated intervention (give reasons) (n=0)

Analysed (n=23)
♦ Excluded from analysis (give reasons) (n=2) – did not perform SMBG in all points as requested

Randomized (n=25)
